# Saturation pulse design for quantitative myocardial T_1_ mapping

**DOI:** 10.1186/s12968-015-0187-0

**Published:** 2015-10-01

**Authors:** Kelvin Chow, Peter Kellman, Bruce S. Spottiswoode, Sonia Nielles-Vallespin, Andrew E. Arai, Michael Salerno, Richard B. Thompson

**Affiliations:** Department of Biomedical Engineering, Faculty of Medicine and Dentistry, 1082 Research Transition Facility, University of Alberta, Edmonton, AB T6G 2V2 Canada; National Institutes of Health, Department of Health and Human Services, National Heart, Lung and Blood Institute, Bethesda, MD USA; Cardiovascular MR R&D, Siemens Medical Solutions USA, Inc., Chicago, IL USA; Cardiovascular Division, Departments of Medicine, Radiology and Medical Imaging, University of Virginia Health System, Charlottesville, VA USA; Biomedical Engineering, University of Virginia Health System, Charlottesville, VA USA

**Keywords:** Saturation pulses, Adiabatic pulses, B_0_ inhomogeneity, B_1_ inhomogeneity, T_1_ mapping, Saturation recovery, SASHA

## Abstract

**Background:**

Quantitative saturation-recovery based T_1_ mapping sequences are less sensitive to systematic errors than the Modified Look-Locker Inversion recovery (MOLLI) technique but require high performance saturation pulses. We propose to optimize adiabatic and pulse train saturation pulses for quantitative T_1_ mapping to have <1 % absolute residual longitudinal magnetization (|M_Z_/M_0_|) over ranges of B_0_ and ${\widehat{B}}_1$ (B_1_ scale factor) inhomogeneity found at 1.5 T and 3 T.

**Methods:**

Design parameters for an adiabatic BIR4-90 pulse were optimized for improved performance within 1.5 T B_0_ (±120 Hz) and ${\widehat{B}}_1$ (0.7–1.0) ranges. Flip angles in hard pulse trains of 3–6 pulses were optimized for 1.5 T and 3 T, with consideration of T_1_ values, field inhomogeneities (B_0_ = ±240 Hz and ${\widehat{B}}_1$=0.4–1.2 at 3 T), and maximum achievable B_1_ field strength. Residual M_Z_/M_0_ was simulated and measured experimentally for current standard and optimized saturation pulses in phantoms and in-vivo human studies. T_1_ maps were acquired at 3 T in human subjects and a swine using a SAturation recovery single-SHot Acquisition (SASHA) technique with a standard 90°-90°-90° and an optimized 6-pulse train.

**Results:**

Measured residual M_Z_/M_0_ in phantoms had excellent agreement with simulations over a wide range of B_0_ and ${\widehat{B}}_1$. The optimized BIR4-90 reduced the maximum residual |M_Z_/M_0_| to <1 %, a 5.8× reduction compared to a reference BIR4-90. An optimized 3-pulse train achieved a maximum residual |M_Z_/M_0_| <1 % for the 1.5 T optimization range compared to 11.3 % for a standard 90°-90°-90° pulse train, while a 6-pulse train met this target for the wider 3 T ranges of B_0_ and ${\widehat{B}}_1$. The 6-pulse train demonstrated more uniform saturation across both the myocardium and entire field of view than other saturation pulses in human studies. T_1_ maps were more spatially homogeneous with 6-pulse train SASHA than the reference 90°-90°-90° SASHA in both human and animal studies.

**Conclusions:**

Adiabatic and pulse train saturation pulses optimized for different constraints found at 1.5 T and 3 T achieved <1 % residual |M_Z_/M_0_| in phantom experiments, enabling greater accuracy in quantitative saturation recovery T_1_ imaging.

**Electronic supplementary material:**

The online version of this article (doi:10.1186/s12968-015-0187-0) contains supplementary material, which is available to authorized users.

## Background

Saturation pulses are commonly used in MRI pulse sequences to prepare longitudinal magnetization to a known zero state, independent of previous acquisitions, and generate spin–lattice (T_1_) relaxation contrast. Several methods for T_1_ quantification in the heart are based on multiple image acquisitions with variable saturation recovery times such as the saturation recovery turbo flash (SRTFL) [1], short acquisition period (SAP-T1) [2], and the recently proposed SAturation recovery single-SHot Acquisition (SASHA) [3] sequences.

All of these techniques assume ideal saturation efficiency, and poor saturation performance results in errors in calculated T_1_ values. The saturation efficiency can be quantified using residual longitudinal magnetization (M_Z_/M_0_), which can range from −1 (full inversion) to 1 (no effect) and 0 for perfect saturation. In the standard three-parameter exponential model for SASHA T_1_ data, saturation efficiency is a fitted parameter, but imperfect saturation may still result in errors in the calculated T_1_ values due to residual magnetization remaining between heartbeats [3]. Two-parameter fitting of SASHA data assuming ideal saturation has been shown to substantially reduce variability of calculated T_1_ values [4], but results in larger T_1_ errors when there is imperfect saturation.

Composite saturation pulses consisting of trains of shaped RF pulses with numerically optimized flip angles have been designed for several different ranges of B_0_ (off-resonance) and ${\widehat{B}}_1$ scale factors (ratio of actual radiofrequency field strength to nominal radiofrequency field strength). For example, enhanced water suppression has been achieved over narrow B_0_ and ${\widehat{B}}_1$ ranges for spectroscopy applications at 1.5 T [5] and numerically optimized adiabatic pulses have been used for wide B_0_/${\widehat{B}}_1$ ranges at 7 T [6]. Hard RF pulse trains have also been proposed [7] and a train of 3 pulses with flip angles tailored for improved performance over B_0_ and ${\widehat{B}}_1$ ranges expected at 3 T has been evaluated in-vivo [8]. However, the maximum residual M_Z_/M_0_ of >8 % of this design may be a significant source of error when used in quantitative imaging sequences.

Adiabatic BIR4-90 saturation pulses [9] and hybrid adiabatic-rectangular saturation pulses [10] have also been proposed, but the high B_1_ field strength required to meet the adiabatic criteria may exceed the allowable specific absorption rate (SAR), particularly on larger subjects. On higher B_0_ field strength magnets, lower ${\widehat{B}}_1$ scale factors may also reduce the effective B_1_ field to below the adiabatic limit and result in poor performance [8, 11].

In this work, we expand upon existing literature regarding the design of saturation pulse trains and adiabatic saturation pulses with the primary goal of significantly improving performance to less than 1 % residual |M_Z_/M_0_| over ranges of B_0_ and ${\widehat{B}}_1$ values found at 1.5 T and 3 T, given the practical constraints of peak B_1_ transmit field strength. The performance of the proposed saturation pulses are simulated, experimentally validated, and compared to current standard saturation pulses in phantom experiments and in-vivo human studies. The effect of saturation pulse performance on the two-parameter SASHA T_1_ mapping technique is also characterized through simulations and in-vivo studies on humans and a swine.

## Methods

### B_1_ field strength limitations

The maximum B_1_ field strength is predominately limited by the achievable RF amplifier voltage for a given RF coil. These B_1_ limitations reduce the off-resonance performance of both adiabatic pulses and hard pulse trains, imposing an additional constraint in their optimization. For the MRI systems used in this study, a reference voltage was calculated for each subject corresponding to the amplifier voltage required to achieve a B_1_ field strength of 11.7 μT, equivalent to a 180° flip angle with a 1 ms rectangular pulse. The maximum achievable B_1_ was calculated by multiplying 11.7 μT by the ratio of maximum voltage to the reference voltage. The maximum achievable B_1_ field strength was calculated in this way for subjects on Siemens MAGNETOM 1.5 T Aera and 3 T Skyra systems over an approximate period of 6 months, with information collected as quality assurance data and analysis approved by the NIH Office of Human Subject Research.

### Spoiler gradient design

**Fig. 1:**
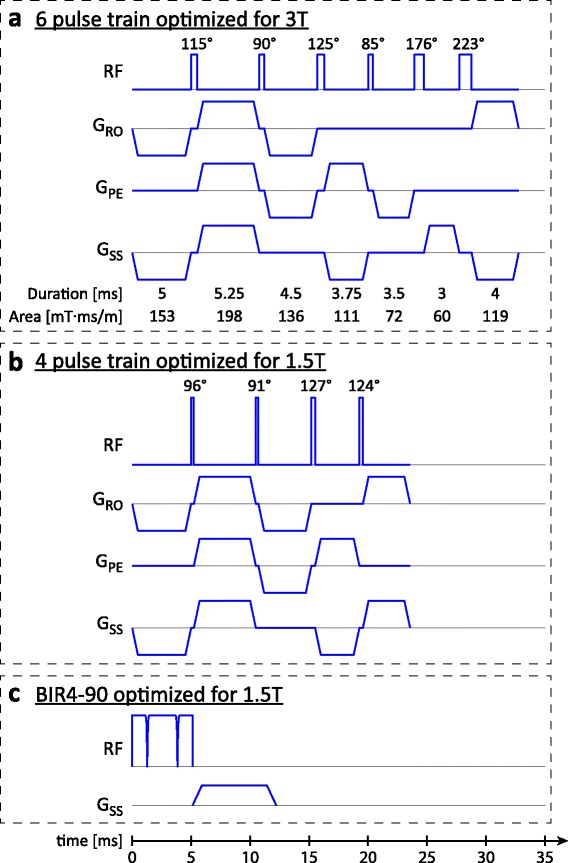
**a**. 6 pulse train numerically optimized for 3 T (${\widehat{B}}_1$ = 0.4–1.2, B_0_ = ±240 Hz, B_1_ field = 14 μT), with a total duration of 32.8 ms. Gradient areas are the effective areas across all three gradient directions. **b**. 4 pulse train numerically optimized for 1.5 T (${\widehat{B}}_1$ = 0.7–1.0, B_0_ = ±120 Hz, B_1_ field = 26.9 μT), with a total duration of 23.6 ms. The first four spoilers are the same for the 4 and 6 pulse trains and the last spoilers have the same pattern, but with the polarity in the slice select axis reversed to maintain alternating polarity. **c**. The optimized BIR4-90 pulse, with a total duration of 12.2 ms. All saturation pulses are shown on the same time, RF amplitude and gradient strength scale

Spoiler gradients in between RF pulses in a saturation pulse train were designed to minimize transverse magnetization. In contrast to previous pulse train designs where inter-pulse spoilers were polarity cycled along a single direction [7, 8, 10], inter-pulse spoilers in the proposed design were cycled along multiple gradient directions in order to dephase magnetization in all spatial directions and reduce the likelihood that spoiler gradients could be unwound during image readouts. Spoiler durations and total areas were also varied to minimize the potential formation of coherent stimulated echoes, with no two spoilers on the same axis having the same area. The implementation in this study uses a gradient strength of 24 mT/m per channel, which is achievable on most modern MRI scanners. At this strength, the minimum spoiler area in the 6-pulse train was 60 mT⋅ms/m, equivalent to 2π phase dispersion across 0.4 mm, with other spoiler areas and directions detailed in Fig. 1a. Spoiler designs for shorter pulse trains with *n* pulses share the same design as the first *n* spoiler gradients in the 6 pulse design, as shown for a 4-pulse train in Fig. 1b. The final trailing spoiler gradients are also of the same design, although its polarity is adjusted to maintain alternating polarity within that axis.

### Numerical pulse train design

The residual M_Z_/M_0_ following pulse train saturation is dependent on the flip angle order due to T_1_ effects and thus direct gradient descent minimization of an ordered flip angle set may be trapped in local minima. Instead, an unordered set of flip angles was minimized using a standard Nelder-Mead algorithm [12], but in each iteration, all possible permutations of the flip angle set were evaluated and the lowest |M_Z_/M_0_| from all permutations was returned. A detailed description of the minimization algorithm can be found in Additional file 1 and MATLAB source code used is provided in Additional files 2, 3, 4, 5 and 6 and available online at https://bitbucket.org/kelvinc/pulsetrainopt.

Optimal flip angles were calculated for pulse trains ranging from 3 to 6 RF pulses. Pulse trains were optimized for 1.5 T using a ${\widehat{B}}_1$ range of 0.7–1.0 [13], a B_0_ range corresponding to ±120 Hz, and a maximum B_1_ field strength of 26.9 μT. Pulse trains were also optimized for 3 T using a larger ${\widehat{B}}_1$ range of 0.4–1.2 [8, 14], a B_0_ range corresponding to ±240 Hz, and a maximum B_1_ of 14 μT. In all Bloch simulation optimizations, the T_1_ range was 200–2000 ms and T_2_ was 45 ms (native myocardium). T_2_ dependence of optimized pulse trains was characterized through simulations using T_2_ values from 5 ms to 250 ms.

Proposed saturation pulse trains were compared to a commonly used reference pulse train consisting of three 90° RF pulses, each with a fixed duration of 0.5 ms [7]. Spoiler durations for the reference pulse train were 1.00, 8.80, 5.86, and 1.55 ms, with a total time of 18.71 ms.

### BIR4-90 pulse optimization

A BIR4-90 pulse was numerically optimized by a brute force optimization over adiabatic design parameters [15]. The pulse duration, amplitude, maximum frequency sweep, and parameters ζ and κ which define the adiabatic half passage section of the BIR4-90 defined in Equations 4 and 6 in [15] were varied and the optimum pulse was selected which achieved the best saturation over the specified range of ${\widehat{B}}_1$ and off-resonance, i.e., best worst-case deviation. The frequency sweep parameter was varied from 4–20 kHz in 1 kHz steps, tan(κ) ranged from 8 to 22 in steps of 2, and ζ ranged from 8 to 30 in steps of 2. Bloch simulations assumed values for native myocardium of T_1_ = 1100 ms and T_2_ = 45 ms. Pulse durations between 4 and 10 ms were evaluated. The design range was ±120 Hz off-resonance and ${\widehat{B}}_1$ ranged from 0.7 to 1.0. The peak amplitude was limited to approximately 20 μT. The optimized BIR4-90 pulse was further characterized with simulations using T_2_ values from 5 ms to 250 ms.

### RF power calculation

Relative RF energy was calculated for each saturation pulse as B_1_^2^ integrated over time divided by the RF energy of the 90°-90°-90° pulse train. Relative peak RF power was calculated as the maximum B_1_^2^ for each saturation pulse, normalized to the maximum B_1_^2^ in the reference 90°-90°-90° pulse train.

### Phantom validation

Saturation efficiency was experimentally assessed for five saturation pulses: the reference 90°-90°-90° pulse train, the 3 T optimized 6-pulse train (Fig. 1a), the 1.5 T optimized 4-pulse train (Fig. 1b), the reference BIR4-90 pulse, and the optimized BIR4-90 pulse (Fig. 1c). Performance for each pulse was assessed in a Siemens 2 L plastic bottle phantom (1.25 g NiSO_4_⋅6H_2_O + 5 g NaCl per 1000 g water). Imaging was performed on a Siemens 1.5 T MAGNETOM Aera MRI scanner (Siemens Healthcare; Erlangen, Germany) and the pulses in the 3 T 6-pulse train were limited to 14 μT to reflect the typical limitation at 3 T. The T_1_ value was measured using an inversion recovery spin echo sequence with inversion times between 100 ms and 500 ms and a non-prepared image. T_2_ was measured using spin echo repeated with echo times varied between 11 ms and 300 ms. All spin echo imaging was performed with a single k-space line acquired per excitation and a 10 s TR to ensure complete magnetization recovery between excitations.

A linear spatial gradient was applied along the long axis of the phantom to assess performance as a function of off-resonance. To calculate an off-resonance (B_0_) map, gradient echo images were acquired with 5 echo times (TE) between 4 and 6 ms, 3000 ms repetition time (TR), 90° flip angle, 128 × 44 matrix size, 400 × 138 mm^2^ field of view, and 8 mm slice thickness. The B_0_ map was generated by fitting the equation φ = ΔB_0_⋅TE for every pixel, where φ is the phase of gradient echo images. To calculate an excitation ${\widehat{B}}_1$-field map for the phantom, gradient echo images were acquired with 45°, 60°, 90°, and 120° flip angles, 3.79 ms TE, and other parameters as above. The signal intensity as a function of flip angle was simulated using a Bloch equation simulation accounting for slice profile effects [16], and a ${\widehat{B}}_1$ map was generated by fitting the measured signal intensity to this simulated curve for every pixel.

Saturation pulse efficiency was calculated using a gradient echo sequence with 3.79 ms TE, 3000 ms TR, 90° flip angle, and other parameters as above. Images were acquired without a saturation pulse and with 4 saturation recovery times (TS = 10–40 ms) for each of the 5 saturation pulses, where TS is defined as the time from the end of the last RF pulse in the saturation and the middle of the imaging excitation RF pulse. A saturation efficiency map was generated by fitting acquired data to {Signal = A*[1 - ηexp(−TS/T_1_)]}, where η is the saturation efficiency and residual (signed) M_Z_/M_0_ is equal to 1-η. These acquisitions were repeated 9 times with the flip angles of the saturation RF pulses scaled by 40–120 % to emulate the effect of varied ${\widehat{B}}_1$ values.

For each pixel of the nine resulting residual M_Z_/M_0_ maps, the B_0_ and effective ${\widehat{B}}_1$ (equal to ${\widehat{B}}_1$ map multiplied by the flip angle scaling factor) could be determined, separately for each saturation pulse considered. Together, the intrinsic variation in ${\widehat{B}}_1$ over the phantom in combination with the 9 repeated experiments with flip angle scaling from 40 % to 120 % yielded a wide range of effective ${\widehat{B}}_1$ values. With the variation in B_0_ provided by the linear gradient, the M_Z_/M_0_ values were thus directly measured over a wide range of B_0_ and ${\widehat{B}}_1$ values. The missing data within the B_0_–${\widehat{B}}_1$ space was calculated by fitting a surface over the scattered points with a modified ridge estimator using the gridfit function [17].

### SASHA sequence simulation

Bloch equation simulations of the SASHA sequence were performed with a range of residual M_Z_/M_0_ (−5 % to 5 %) and relaxation values emulating myocardium (native T_1_/T_2_: 1175/50 ms, post-contrast T_1_/T_2_: 725/50 ms) and blood (native T_1_/T_2_: 1650/240, post-contrast: 500/180 ms) at 1.5 T [3]. Typical in-vivo SASHA acquisition parameters were used: 1.3/2.6 ms TE/TR, 70° target flip angle, 70 phase encode lines, 60 bpm simulated heart rate, 10 images with maximum 775 ms saturation recovery time (TS) and a non-saturated image. Image readout flip angles were scaled using a variable flip angle (VFA) scheme to minimize T_1_ error with two parameter fitting as previously described [18]. In this scheme, the prescribed flip angle of the first 45 RF pulses was scaled by sin(*x*) for π/90 < *x* < π/2. Simulations used actual RF pulse waveforms and the transverse magnetization (M_XY_) at the centre k-space readout as the image signal intensity. T_1_ values were calculated by fitting simulated data to a 2-parameter exponential recovery model with assumed ideal saturation.

### In vivo human study

Saturation efficiency was assessed in 3 human volunteers on a Siemens 3 T MAGNETOM Skyra MRI scanner (Siemens Healthcare; Erlangen, Germany) using a single-shot centrically ordered gradient echo sequence. Volunteers provided written informed consent and the study was approved by the University of Virginia Health System Institutional Review Board. Typical sequence parameters were: 1.14/2.31 ms TE/TR, 10° flip angle, 128 × 90 matrix, 400 × 281 mm^2^ field of view, and 10 mm slice thickness. Saturation recovery images were acquired for the 90°-90°-90°, 1.5 T 4-pulse, 3 T 6-pulse, reference BIR4-90, and optimized BIR4-90 saturation pulses with a 7.5 ms saturation recovery time, defined from the end of the last saturation RF pulse to the center of the first imaging RF pulse. A proton density image was acquired with the same pulse sequence parameters. All images were obtained in short-axis and 4-chamber orientations, acquired in diastasis with end-expiratory breath-holds.

Saturation recovery images were processed using a phase-sensitive reconstruction using the proton density image phase as a reference in order to avoid Rician noise bias. The residual M_Z_/M_0_ was calculated as the ratio of the saturation recovery image to the proton density image.

${\widehat{B}}_1$ maps were acquired using a free-breathing saturated double angle method [11] with single-shot echo planar imaging (EPI) readouts. Typical sequence parameters were: electrocardiogram (ECG) gated diastasis imaging, 45°/90° flip angles, 80 × 50 matrix, rate 2 parallel imaging (GRAPPA), 360 × 225 mm^2^ field of view, 10 mm slice thickness, 2 saturation bands on the chest and back to reduce the field of view, and 15 repetitions per flip angle. All images were registered and a ${\widehat{B}}_1$ scale factor map was calculated as ${\widehat{B}}_1={ \cos}^{-1}\left(\left|\frac{I_{90{}^{\circ}}}{2{I}_{45{}^{\circ}}}\right|\right)/45{}^{\circ}$, where I_45°_ and I_90°_ are the signal intensities from the 45° and 90° flip angle images respectively.

T_1_ maps were collected using SASHA with 90°-90°-90°, optimized BIR4-90, and 6-pulse saturation and MOLLI with an optimized adiabatic inversion pulse [13] in diastasis with end-expiratory breath-holds. SASHA sequences used a 70° imaging flip angle with VFA modulation and 10 images with the longest TS time allowable by the heart rate [19] and MOLLI used a 35° imaging flip angle with a 5(3)3 sampling pattern.

### In vivo swine study

Myocardial T_1_ mapping was performed on a 66 kg swine on a Siemens 3 T Skyra MRI scanner using the SASHA and MOLLI [20] T_1_ mapping sequences. SASHA was acquired using both 90°-90°-90° and 6-pulse saturation with a 45° imaging flip angle and VFA modulation. MOLLI used an optimized inversion pulse with a 20° imaging flip angle. A ${\widehat{B}}_1$ map was acquired using a saturated double angle method as described above. Swine were studied in accordance with a protocol approved by the Animal Care and Use Committee of the National Institutes of Health (protocol number H-0214).

## Results

### B_1_ field strength limitations

The maximum B_1_ field strength was calculated for 379 subjects on the Siemens MAGNETOM Skyra platform and 230 subjects on the Siemens MAGNETOM Aera platform. A maximum B_1_ field strength of 14 μT was achievable on the 3 T Skyra platform in >98 % of subjects, while 26.9 μT was achievable on the 1.5 T Aera platform in >99 % of subjects. At a B_1_ strength of 14 μT, a 90° rectangular pulse is 419 μs and the full width half maximum effective B_1_ scaling is ±1334 Hz.

### BIR4-90 pulses

The numerically optimized BIR4-90 design used a duration of 5.12 ms, maximum B_1_ field of 20.6 μT, swept over ±7 kHz, with ζ = 22 and tan(κ) = 18. This design is longer and has a lower maximum B_1_ compared to the reference BIR4-90 pulse that has a 4.00 ms duration and a maximum B_1_ of 25.1 μT, resulting in lower relative peak RF energy and power (Table 1). The optimized BIR4-90 design had substantially improved performance with a maximum residual |M_Z_/M_0_| within the 1.5 T optimization range 5.8× lower than the reference pulse. More detailed comparisons of the two BIR4-90 pulses are characterized in the phantom study below.

**Table 1:** Summary of characteristics for Bloch simulation numerically optimized saturation pulses

		Flip Angle [°]	Duration [ms]	Relative RF Energy	Relative Peak RF Power	Residual |M_Z_/M_0_| [%] mean±std (max)
Reference saturation pulses	90°-90°-90°	90-90-90	18.7	1.0	1.0	2.86±2.98 (11.31)
	BIR4-90	25.1 μT (adiabatic)	11.1	11.0	4.1	0.92±0.92 (5.32)
Optimized for 1.5T	BIR4-90	20.6 μT (adiabatic)	12.2	10.0	2.9	0.30±0.14 (0.91)
B_0_ = ±120 Hz	3 pulse train	90−107−126	19.5	2.7	5.2	0.40±0.21 (0.80)
${\widehat{B}}_1$=0.7–1.0	4 pulse train	96−91−127−124	23.6	3.7	5.2	0.11±0.07 (0.25)
B_1_ field = 26.9 μT	5 pulse train	89−106−89−145−127	27.4	4.7	5.2	0.06±0.03 (0.11)
	6 pulse train	116−99−81−92−146−139	30.6	5.7	5.2	0.02±0.01 (0.04)
Optimized for 3T	3 pulse train	220−136−93	20.8	2.0	1.4	4.33±2.49 (8.70)
B_0_ = ±240 Hz	4 pulse train	75−103−149−213	25.0	2.4	1.4	1.99±1.22 (4.17)
${\widehat{B}}_1$= 0.4–1.2	5 pulse train	99−76−120−169−220	29.2	3.0	1.4	0.64±0.41 (1.58)
B_1_ field = 14 μT	6 pulse train	115−90−125−85−176−223	32.8	3.6	1.4	0.27±0.19 (0.87)

Total duration values include post-spoiler and pre-spoilers where applicable. Residual M_Z_/M_0_ values are simulated using Bloch equation simulations and the mean, standard deviation, and maximum are calculated over the entire optimization space, i.e. B_0_, ${\widehat{B}}_1$, and T_1_. Residual M_Z_/M_0_ for reference pulses was evaluated over the 1.5 T ${\widehat{B}}_1$ range. Relative RF energy and power are normalized to a reference 90°-90°-90° pulse train as described in the methods

### Numerically optimized pulse trains

**Fig. 2:**
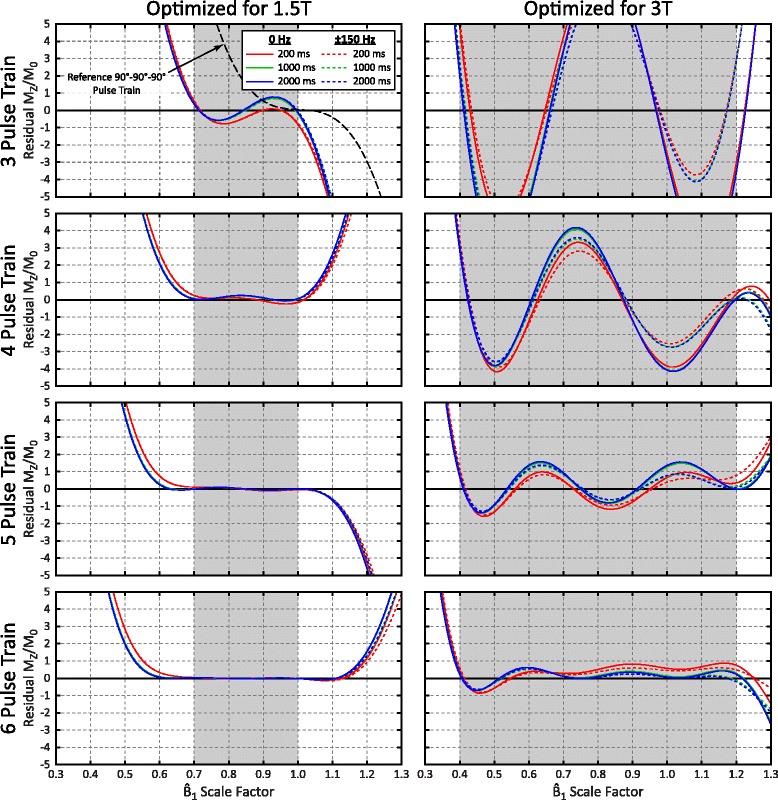
Simulated performance for pulse trains with 3–6 pulses, visualized for 3 T_1_ values (*colored lines*) and off-resonance (B_0_) values (*solid vs. dashed lines*). Performance for the reference 90°-90°-90° pulse train (on-resonance, 1000 ms T_1_) is also shown along with the 3 pulse train optimized for 1.5 T for comparison (*top left*)

Characteristics of the Bloch simulation numerically optimized pulse trains are summarized in Table 1 and their simulated performance is plotted in Fig. 2. The reference 90°-90°-90 pulse train has poor performance at the lower end of ${\widehat{B}}_1$ scale factors expected at 1.5 T, with a residual M_Z_/M_0_ of 10 % at a ${\widehat{B}}_1$ of 0.7 while on-resonance and a 1000 ms T_1_. For the 1.5 T optimization range, the optimized 3-pulse train (90°-107°-126°) achieved the target residual |M_Z_/M_0_| of <1 %, while 6 pulses (115°-90°-125°-85°-176°-223°) were necessary to achieve this target for the 3 T optimization range due to the larger ${\widehat{B}}_1$ range of 0.4–1.2. For both ${\widehat{B}}_1$ optimization ranges, simulated residual |M_Z_/M_0_| increased rapidly outside of the optimization region. Minor off-resonance and T_1_ effects were also observed, but did not have a consistent trend over the range (Fig. 2).

### Pulse train ordering effects

The saturation efficiency of a saturation pulse train is dependent on the order of its flip angles due to T_1_ relaxation. The effect of flip angle order in pulse trains can be illustrated by characterizing the residual |M_Z_/M_0_| for all permutations of a set of flip angles. For the 6-pulse train optimized for 3 T (Table 1), the residual |M_Z_/M_0_| was calculated for all 720 flip angle permutations during the final iteration of the Bloch simulation numerical optimization algorithm. The maximum and mean residual |M_Z_/M_0_| are calculated over the optimization range of B_0_, ${\widehat{B}}_1$, and T_1_ values and plotted (Additional file 7) after sorting by the maximum residual |M_Z_/M_0_|. There was relatively poor correlation between the maximum and mean |M_Z_/M_0_|, as some permutations with similar maximum |M_Z_/M_0_| had greatly different mean |M_Z_/M_0_|. The largest maximum residual |M_Z_/M_0_| was 5.7× greater than the smallest, and the largest mean residual |M_Z_/M_0_| was 2.6× greater than the smallest.

### T_1_ relaxation effects

**Fig. 3:**
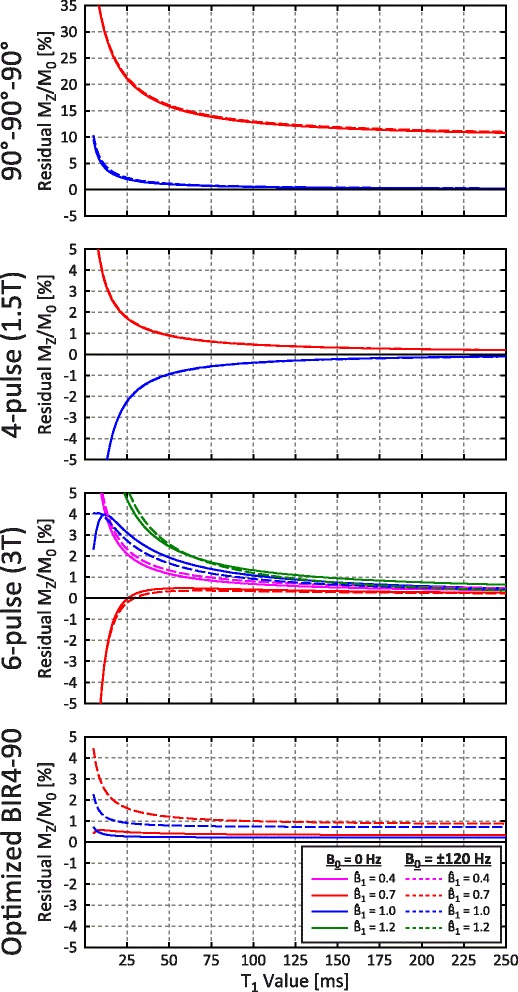
Simulated residual M_Z_/M_0_ for selected saturation pulses as a function of T_1_ for different off-resonance (B_0_) and ${\widehat{B}}_1$ values

Simulated residual M_Z_/M_0_ for selected saturation pulses are shown as a function of ${\widehat{B}}_1$ and T_1_ in Fig. 3. A larger range of ${\widehat{B}}_1$ values (0.4 and 1.2) are shown only for the 6-pulse train, which is full range for the 3 T field strength. Performance is only weakly related to T_1_ in the optimization range used for pulse design (200–2000 ms), but residual |M_Z_/M_0_| increases rapidly for T_1_ values shorter than 25 ms for all pulse trains, with divergent behavior depending on the ${\widehat{B}}_1$ scale factor. The 90°-90°-90° train has poor performance with a ${\widehat{B}}_1$ scale factor of 0.7, with residual |M_Z_/M_0_| exceeding 15 % for a 50 ms T_1_. The 3 T 6-pulse train maintains |M_Z_/M_0_| < 1 % for T_1_ greater than 186 ms at ±240 Hz and for T_1_ greater than 140 ms at ±120 Hz. The optimized BIR4-90 pulse had the best performance at short T_1_ values, with 1.6 % residual |M_Z_/M_0_| at 25 ms.

### T_2_ relaxation effects

**Fig. 4:**
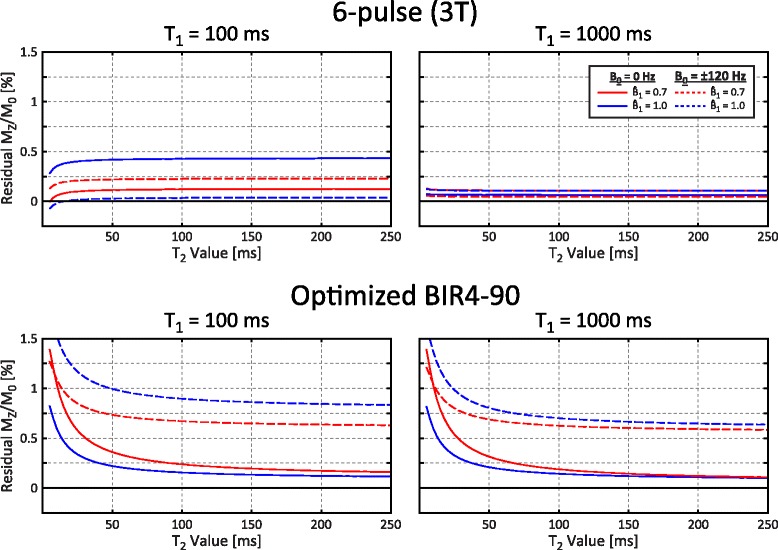
Simulated residual M_Z_/M_0_ for selected saturation pulses as a function of T_2_ for different T_1_, off-resonance (B_0_) and ${\widehat{B}}_1$ values

The effects of T_2_ relaxation on residual M_Z_/M_0_ are shown for the optimized 3 T 6-pulse train and BIR4-90 saturation pulses in Fig. 4. Saturation pulse trains were insensitive to T_2_ relaxation and the optimized BIR4-90 pulse had less than a 1 % change in residual M_Z_/M_0_ for T_2_ values from 25 to 250 ms.

### Phantom validation

**Fig. 5:**
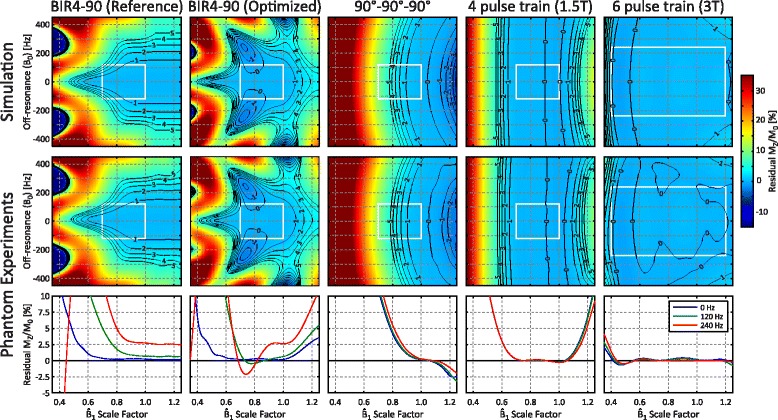
Simulated residual M_Z_/M_0_ (*top row*) and experimentally measured values (*middle row*) for 5 different saturation pulses as a function of ${\widehat{B}}_1$ scale factor and off-resonance. The *bottom row* shows a profile of measured residual M_Z_/M_0_ as a function of ${\widehat{B}}_1$ scale factor for three off-resonance frequencies

The T_1_ and T_2_ of the bottle phantom was measured to be 316 ms and 274 ms respectively using spin echo experiments, and the intrinsic ${\widehat{B}}_1$ scaling factor across the image was found to range from 0.83 to 1.05. The applied linear magnetic field gradient resulted in off-resonance values from −605 to 697 Hz across the phantom. The performance of each saturation pulse as a function of B_0_ and ${\widehat{B}}_1$ scaling factor is plotted in the top row of Fig. 5, with the optimization range of B_0_ and ${\widehat{B}}_1$ values demarcated with a white box.

Experimentally measured residual M_Z_/M_0_ (middle row, Fig. 5) shows excellent agreement with simulations (top row, Fig. 5). Saturation pulse trains are robust to off-resonance due to the short individual pulse durations, while the BIR4-90 pulses with an overall shorter duration have tighter off-resonance constraints and more complex relationships with B_0_ and ${\widehat{B}}_1$. The reference 90°-90°-90 pulse train shows poor performance at low ${\widehat{B}}_1$ scaling factors, even in the relatively narrow range of 0.7–1.0 expected at 1.5 T. All proposed pulses show greatly improved performance within their respective optimization spaces (white dashed box) compared to the reference designs. The average measured residual |M_Z_/M_0_| within these spaces was 0.24 ± 0.16 %, 0.11 ± 0.06 %, and 0.20 ± 0.18 % for the optimized BIR4-90, 4 pulse (1.5 T), and 6 pulse (3 T) trains respectively.

### SASHA sequence simulation

**Fig. 6:**
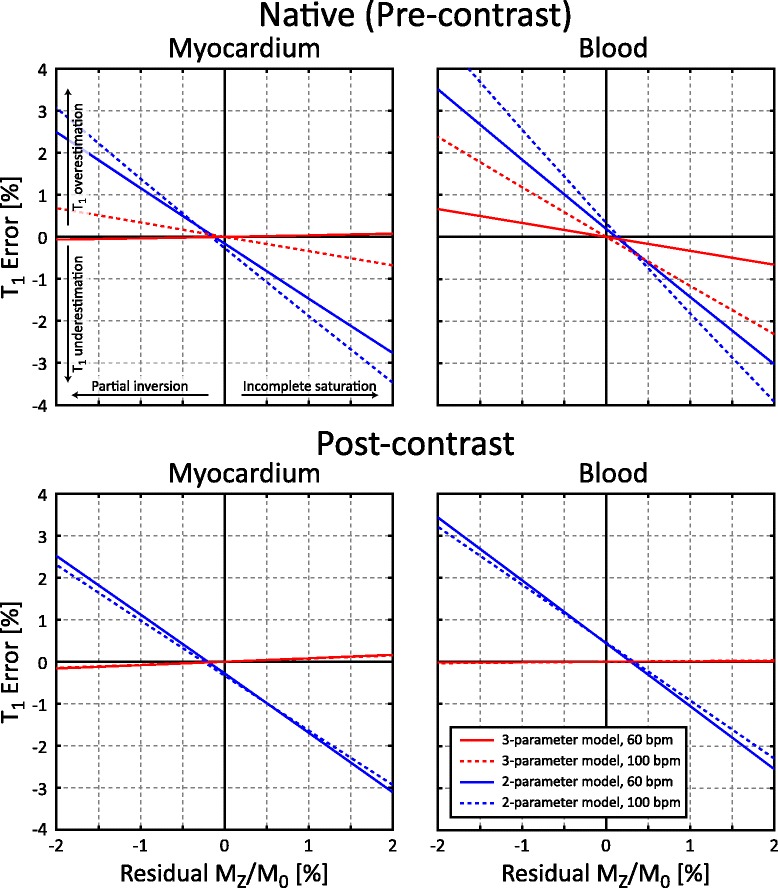
Simulated T_1_ error for SASHA as a function of residual M_Z_/M_0_ for native (*top*) and post-contrast (*bottom*) myocardium (*left*) and blood (*right*) using 2 and 3 parameter models at 60 and 120 bpm. Input native relaxation times were T_1_/T_2_ = 1175/50 ms (myocardium) and T_1_/T_2_ = 1650/240 (blood) and post-contrast relaxation times were T_1_/T_2_ = 725/50 ms (myocardium) and T_1_/T_2_ = 500/180 ms (blood)

Figure 6 shows the relationship between residual M_Z_/M_0_ and simulated T_1_ errors in the SASHA sequence. SASHA T_1_s are generally underestimated with incomplete saturation, with a 1.3–1.6 % increase in 2-parameter SASHA T_1_ error for every 1 % change in residual M_Z_/M_0_. A higher heart rate of 100 bpm results in greater SASHA T_1_ errors for native (pre-contrast) T_1_/T_2_ values compared to 60 bpm, with negligible effects for post-contrast values. Overall, 2-parameter model fitting results in greater T_1_ error dependence on residual M_Z_/M_0_ than 3-parameter fitting, particularly for the lower post-contrast T_1_/T_2_ values.

### In vivo human study

**Fig. 7:**
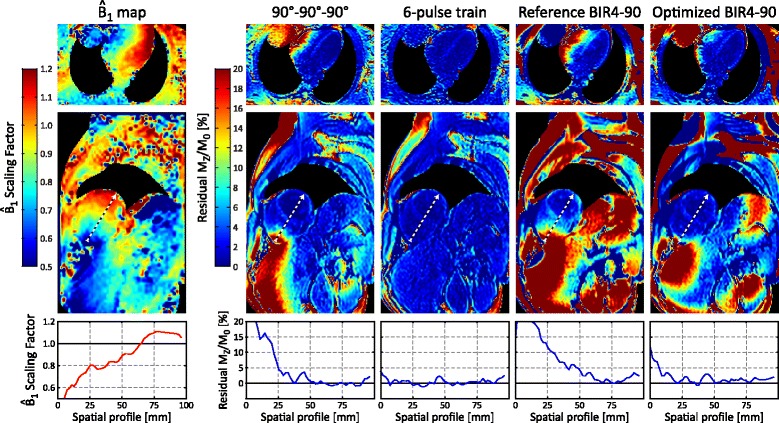
${\widehat{B}}_1$ scale factor and residual M_Z_/M_0_ maps measured in a human volunteer for various saturation pulses. Air regions with low signal intensity are masked for visualization. A profile is extracted from the short axis images (*black and white dashed line*) across both the right and left ventricles

Subjects had an average heart rate of 63 ± 10 bpm with no mis-triggering. Representative ${\widehat{B}}_1$ and residual M_Z_/M_0_ maps from one subject are shown in Fig. 7. ${\widehat{B}}_1$ was decreased near the acute margin of the right ventricle and coincided with increased residual M_Z_/M_0_ values for the 90°-90°-90° and reference BIR4-90 pulses. A similar spatial pattern was observed in all subjects, although there was considerable variability in ${\widehat{B}}_1$ between subjects (Table 2). The optimized BIR4-90 pulse had lower residual M_Z_/M_0_ than the reference BIR4-90 pulse in the myocardium (2.8 ± 1.1 % vs 6.1 ± 3.1 %) as well as in the blood pool. The 6-pulse train had the lowest overall residual M_Z_/M_0_ in the myocardium (1.8 ± 0.3 %) and significantly more uniform saturation over the entire body (Fig. 7).

**Table 2:** Measured residual longitudinal magnetization (M_Z_/M_0_) at 3 T in 3 human subjects

		Residual longitudinal magnetization [%]				
	${\widehat{B}}_1$ scale factor	90°-90°-90°	1.5 T 4-pulse train	3 T 6-pulse train	Reference BIR4-90	Optimized BIR4-90
LV myocardium	0.87 ± 0.21	3.5 ± 2.3	3.4 ± 0.5	1.8 ± 0.3	6.1 ± 3.1	2.8 ± 1.1
LV blood pool	0.94 ± 0.19	0.5 ± 1.1	2.1 ± 1.7	0.6 ± 0.1	2.5 ± 2.3	0.8 ± 0.3
RV blood pool	0.86 ± 0.12	7.5 ± 7.5	0.8 ± 0.5	0.6 ± 0.1	10.6 ± 7.5	2.3 ± 2.2

Calculated in-vivo T_1_ values for the SASHA sequence with various saturation pulses and MOLLI are summarized in Table 3. The 6-pulse SASHA sequence had longer myocardial and blood T_1_ values than the 90°-90°-90° and optimized BIR-4 variants and all SASHA T_1_ values were longer than MOLLI T_1_ values. Right ventricular blood T_1_s were lower than left ventricular blood T_1_s for all sequences, with the largest differences in the 90°-90°-90° SASHA and MOLLI data (193 ± 100 ms and 143 ± 83 ms respectively).

**Table 3:** Measured T_1_ values at 3 T in 3 human subjects

	SASHA T_1_ [ms]			MOLLI T_1_ [ms]
	90°-90°-90°	Optimized BIR4-90	3 T 6-pulse train	
LV myocardium	1431 ± 55	1470 ± 47	1491 ± 30	1212 ± 11
LV blood pool	1921 ± 132	1845 ± 97	2048 ± 69	1811 ± 81
RV blood pool	1728 ± 229	1819 ± 113	1991 ± 57	1668 ± 162
LV - RV blood pool	193 ± 100	26 ± 50	57 ± 13	143 ± 82

### In vivo swine study

**Fig. 8:**
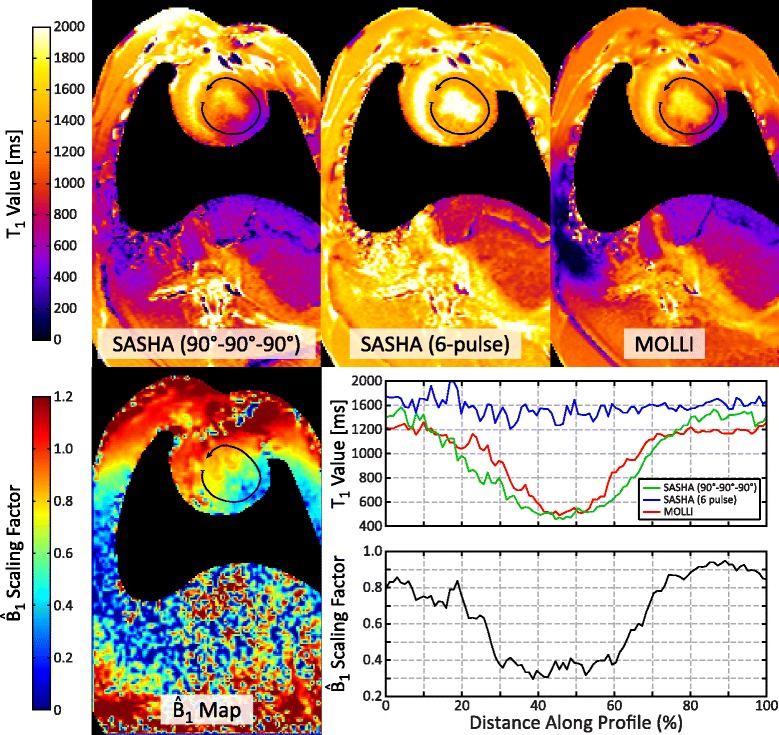
A ${\widehat{B}}_1$ map (*bottom left*) and T_1_ maps (*top row*) using SASHA with a reference 90°-90°-90° saturation pulse, SASHA with a proposed 6-pulse train, and MOLLI in a swine. Air regions with low signal intensities are masked for visualization. A profile of T_1_ values along the left ventricular wall shows decreased T_1_ values in the lateral wall coinciding with reduced ${\widehat{B}}_1$ values

Parametric T_1_ and ${\widehat{B}}_1$ maps acquired in a swine are shown in Fig. 8 and a profile is extracted along the left ventricle. Myocardial T_1_ values from the 90°-90°-90° SASHA and MOLLI sequences show a >50 % artifactual decrease in the lateral wall, spatially coinciding with reduced ${\widehat{B}}_1$ values. T_1_ values using the 6-pulse SASHA sequence were more spatially uniform across both the myocardium (1386 ± 70 ms along the profile) and the overall field of view.

## Discussion

In this study, adiabatic BIR4-90 and pulse train saturation designs were optimized for 1.5 T and 3 T with the objective of achieving <1 % residual |M_Z_/M_0_| for the wide ranges of ${\widehat{B}}_1$ and B_0_ inhomogeneities expected at these field strengths, while imposing the typical peak B_1_ field amplitude limitations measured on 1.5 T and 3 T scanners. New pulses were shown to have significantly improved saturation performance as compared to current standard pulses. Measured saturation performance in phantoms had excellent agreement with simulations and in-vivo experiments demonstrated the superior performance of the proposed 6-pulse train saturation pulse. T_1_ maps calculated using SASHA with proposed 6-pulse train saturation showed greater spatial homogeneity than the reference pulse in both human and swine studies.

### BIR4-90 pulse design

Experimental data in this study shows the reference BIR4-90 saturation pulse has relatively poor performance with low ${\widehat{B}}_1$ and high off-resonance within the range expected at 1.5 T. A new optimized BIR4-90 pulse was shown to have improved performance with <1 % residual |M_Z_/M_0_| over the 1.5 T optimization range of B_0_ and ${\widehat{B}}_1$ fields while reducing maximum B_1_ field strength and resulting SAR. Although the required B_1_ of 20.6 μT was achievable on the 1.5 T MRI systems used here, the 14 μT B_1_ limit at 3 T reduces the effective flip angle and saturation performance. In the 3 T human study, the optimized BIR4-90 SASHA had 1.6 % shorter myocardial T_1_ values than 6-pulse SASHA, consistent with T_1_ underestimation from slightly larger residual M_Z_/M_0_.

### Pulse train design

The ordering of flip angles in saturation pulse trains was found to be important, with a reduction in the maximum residual |M_Z_/M_0_| by a factor of 5.7× from the best order permutation to the worst. The evaluation of all permutations of an unordered flip angle set in the minimization subroutine provided a straightforward means of considering flip angle ordering while avoiding trapping within local minima. This is likely to provide a more optimal solution than the previous minimization approach which considered ordering only in the final stage [8].

Pulse trains were designed for expected ranges of ${\widehat{B}}_1$ values at both 1.5 T and 3 T, with excellent performance within those optimization ranges when a sufficient number of pulses are used. As performance decreases rapidly outside of the ${\widehat{B}}_1$ optimization range for all pulse trains (Fig. 2), conservative estimates of ${\widehat{B}}_1$ ranges were used in the design process. Experimentally measured residual M_Z_/M_0_ in a phantom closely matched simulations for both the adiabatic BIR4-90 pulses as well as all saturation pulse trains, confirming the validity of the Bloch equation simulations used in the design process.

For the ${\widehat{B}}_1$ range of 0.7 to 1.0 expected for 1.5 T, the optimized 3-pulse train has the same number of pulses as the reference 90°-90°-90° pulse train and reduces the maximum residual |M_Z_/M_0_| by 14×, meeting the target of <1 %. The 4-pulse train has even better performance with <0.25 % peak residual magnetization (0.11 % mean residual signal), with minimal additional time and SAR.

The design ${\widehat{B}}_1$ range for 3 T was chosen to be 0.4–1.2 to encompass the widest range of in-vivo cardiac ${\widehat{B}}_1$ values measured in a handful of small studies [8, 11, 14, 21]. The in-vivo ${\widehat{B}}_1$ maps for the 3 human subjects in this study show a smaller range of ${\widehat{B}}_1$ values than previous studies, but with a similar spatial pattern of variation primarily in the anteroposterior direction and larger ${\widehat{B}}_1$ values in the lateral wall of the left ventricle [14]. This may be due to a difference in body coil design between scanner manufacturers, and further variations may exist between models with different bore sizes. However, ${\widehat{B}}_1$ values less than 0.4 were observed in the swine model, suggesting that the design ${\widehat{B}}_1$ range may need to be further widened for large animal model studies. Further work is needed to characterize the ranges of ${\widehat{B}}_1$ values in larger representative populations of both human and swine on different scanner platforms.

The 3 T 6-pulse train achieved <1 % peak residual magnetization over the wide design range of ${\widehat{B}}_1$ values, with excellent performance (mean residual |M_Z_/M_0_| of 0.27 %) and only 1.4× greater peak RF power compared to the 90°-90°-90° reference pulse train. The relatively short RF pulse durations used in the pulse trains resulted in a wide range of B_0_ insensitivity for all pulse train designs, with a negligible drop in measured performance at ±450 Hz (Fig. 5). This is consistent with superior saturation of fat signals, which have a ~440 Hz shift in resonant frequency at 3 T, and more uniform saturation over the entire field of view compared to the BIR4-90 pulses (Fig. 7).

### Further pulse train optimization

The saturation pulses presented in this study were optimized for myocardial T_1_ mapping, but their excellent performance over a typical optimization range makes them suitable for other applications as well such as myocardial first-pass perfusion sequences or the saturated double angle method. In settings with much different criteria, such as B_1_ amplitude limitations or B_0_, ${\widehat{B}}_1$, and T_1_ ranges, the supplied optimization code may be used to design more application-specific pulse trains.

For example, while relatively short RF pulses were selected to maximize the off-resonance performance, pulse lengths may be increased considerably to reduce peak B_1_ amplitude and SAR. Additionally, while the maximum B_1_ amplitude was characterized for 2 common MRI scanner models, other models and scanners by different vendors likely have different limitations. The inter-pulse spoiler durations could also be shortened to further reduce T_1_ dependencies, which would improve performance for very short T_1_ values encountered in contrast-enhanced first-pass myocardial perfusion imaging.

### Saturation performance and SASHA T_1_ mapping

Simulations of 2-parameter fitting with SASHA data show increased sensitivity to saturation pulse performance compared to 3-parameter fitting. In human studies, there was consistently lower ${\widehat{B}}_1$ in the right ventricle leading to increased residual longitudinal magnetization with the 90°-90°-90° saturation pulse, but not the 6-pulse train. Correspondingly, right ventricular T_1_ values with 90°-90°-90° SASHA were substantially lower (193 ± 100 ms) than in the left ventricle, but the difference was much lower (57 ± 13 ms) with 6-pulse SASHA which had more spatially uniform saturation. This remaining difference between the left and right ventricular blood with 6-pulse SASHA is comparable to a difference of ~80 ms between atrial and venous blood with a 0.42 hematocrit previously reported at 3 T [22]. Shorter MOLLI T_1_ values compared to SASHA are consistent with known influences of factors such as T_2_, magnetization transfer, and off-resonance on the MOLLI sequence [4].

Substantially larger ${\widehat{B}}_1$ variations observed in the swine may be the result of the more cylindrical chest geometry compared to humans. These variations resulted in a >50 % artifactual decrease in myocardial T_1_ values for both the 90°-90°-90° SASHA and MOLLI sequences while 6-pulse SASHA had spatially homogenous T_1_s. SASHA with a 6-pulse saturation may provide more reliable T_1_ measurements in pre-clinical large animal studies at 3 T, but further study is needed to determine the impact on the quantification of myocardial fibrosis.

### Study limitations

Gradient spoiler design and its effect on the possible formation of stimulated echoes were not studied in detail. The proposed spoiler design utilizes relatively large gradient areas played in multiple directions, with care taken to avoid duplicate gradient areas. While no stimulated echo artifacts were observed in our data, a more systematic study of stimulated echo formation is warranted.

Residual M_Z_/M_0_ was measured in-vivo using images with a saturation recovery time of 7.5 ms, which was the minimal duration due to the length of post-saturation spoiler gradient for the reference BIR4-90 pulse. Calculated residual M_Z_/M_0_ values are likely to have a small positive bias due to T_1_ recovery during this duration. Although centric ordering was used, T_1_ recovery during the readout results in a further positive bias and may explain the larger residual M_Z_/M_0_ in the LV myocardium compared to the blood pool it encloses.

In-vivo measurements in this study were limited to 3 human subjects and a single animal. However, the magnitude and spatial pattern of ${\widehat{B}}_1$ heterogeneity in the human subjects are consistent with existing literature [14] and ${\widehat{B}}_1$ variations in the swine were representative of the authors’ previous experience.

## Conclusions

This study has described the optimization of saturation pulses and validated their performance in both phantom experiments and in-vivo studies. While an optimized BIR4-90 design and a 3-pulse train were able to meet the design criteria of <1 % residual |M_Z_/M_0_| at 1.5 T, only the optimized 6-pulse train met the criteria for B_0_ and ${\widehat{B}}_1$ values expected at 3 T. The robust performance of optimized saturation pulse trains to a wide range of off-resonance, T_1_ values, and ${\widehat{B}}_1$ scale factors can minimize these as sources of errors in quantitative saturation-recovery based sequences such as SASHA or in other applications such as first pass perfusion imaging.

## Additional files

Additional file 1:
**Numerical pulse train optimization algorithm.** (DOCX 137 kb) Additional file 2:
**Main optimization code (MATLAB).** (TXT 5 kb) Additional file 3:
**Additional optimization code (MATLAB).** (TXT 2 kb) Additional file 4:
**Additional optimization code (MATLAB).** (TXT 2 kb) Additional file 5:
**Additional optimization code (MATLAB).** (TXT 2 kb) Additional file 6:
**Additional optimization code (MATLAB).** (TIFF 4449 kb) Additional file 7:
**Saturation Pulse Performance with Flip Angle Permutations (3 T** **6-pulse** **train).** (TXT 8 kb)

## Abbreviations

ECG: Electrocardiogram
GRAPPA: Generalized auto-calibrating partially parallel acquisition
LV: Left ventricle
MOLLI: Modified Look-Locker inversion recovery
RF: Radiofrequency
RV: Right ventricle
SAR: Specific absorption rate
SASHA: Saturation recovery single-shot acquisition
TE: Echo time
TR: Repetition time
TS: Saturation recovery time
VFA: Variable flip angle
