# Supplementary material for: Saturation pulse design for quantitative myocardial T1 mapping
Source: J Cardiovasc Magn Reson. 2015 Oct 1;17:84. doi: 10.1186/s12968-015-0187-0 (PMC4589956; doi:10.1186/s12968-015-0187-0)
Supplement: Additional file 1: — Numerical pulse train optimization algorithm. (DOCX 137 kb) [file 12968_2015_187_MOESM1_ESM.docx]

**Additional File 1: Numerical Pulse Train Design Algorithm**

Optimal flip angles were calculated using a three-stage minimization algorithm, where flip angles calculated in each stage are used as the initial guess for the next stage in order to minimize the possibility of trapping in local minimia.

In the first stage, the approximate optimal flip angles in a pulse train were algebraically calculated using a geometric model which approximates each pulse as saturating a narrow “bands” of $\hat{B}_{1}$ values where the effective flip angle is close to 90°. The approximate optimal flip angles are such that these bands are equally sized and equally spaced.

The simplest saturation pulse is a single hard RF pulse with a prescribed 90º flip angle. Complete saturation is achieved in regions with perfect B_0_ and B_1_ field calibration, but incomplete saturation or partial inversion results otherwise. Assuming on-resonance, the residual M_Z_/M_0_ for a single hard pulse can be described more generally as $cos\left( \hat{B}_{1}\theta\right)$, where $\hat{B}_{1}$ is a normalized scale factor and θ is the flip angle. Complete saturation occurs when $\hat{B}_{1}\theta$ is equal to 90º or at $\hat{B}_{1}={90^{\circ}}/\theta$. By approximating the cosine function as linear around the null, the maximum residual |M_Z_/M_0_| can be intuitively minimized over a range of $\hat{B}_{1}$ values by minimizing the maximum absolute height of two triangles (Figure below). By geometric inspection, this occurs when the null point (a) is in the middle of the optimization range. Therefore, the optimal flip angle to minimize the peak residual |M_Z_/M_0_|for a range of $\hat{B}_{1}$ values from $\hat{B}_{1min}$ to $\hat{B}_{1max}$ is $\theta_{opt}\approx{180^{\circ}}/\left( \hat{B}_{1min}+\hat{B}_{1max} \right)$.


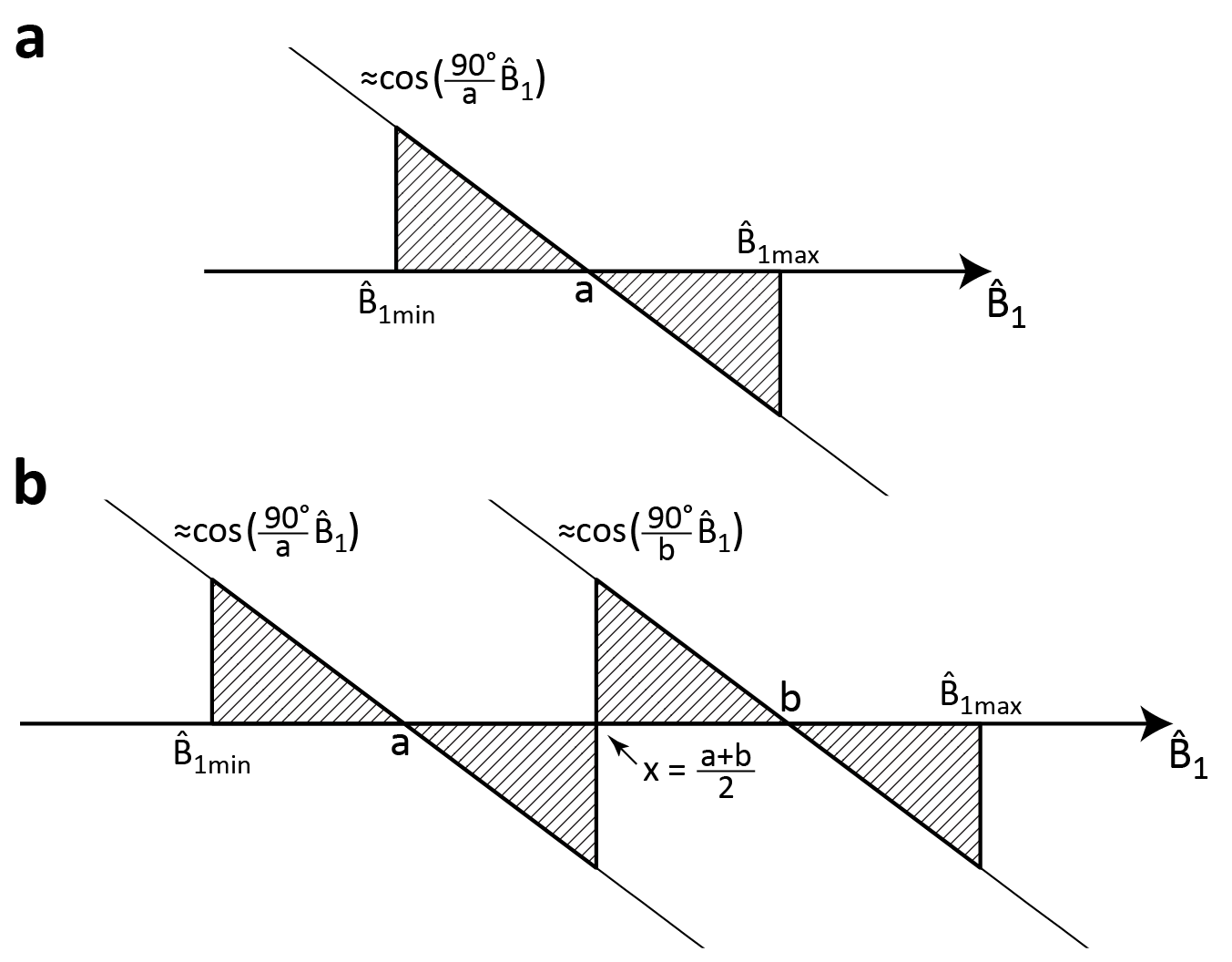


**Figure.** Geometric approach to pulse train design for (**a**) a single RF pulse and (**b**) two RF pulses.

This geometric minimization model can be generalized to multiple hard pulses when the residual M_Z_/M_0_ at a given $\hat{B}_{1}$ is described only by the minimum absolute valued cosine function at that location (Fig. 1b), i.e. ignoring the multiplicative effect of multiple overlapping cosines. Again by geometric inspection, the minimum peak absolute height for all 4 triangles occurs when the null points are at $a=\frac{3}{4}\hat{B}_{1min}+\frac{1}{4}\hat{B}_{1max}$ and $b=\hat{B}_{1max}-\frac{1}{4}\left( \hat{B}_{1max}-\hat{B}_{1min} \right)$. More generally, the optimal solution for *n* RF pulses is when the null points are evenly spaced with distance ${d=\left( \hat{B}_{1min}+\hat{B}_{1max} \right)}/n$, with the first and last null points spaced $d/2$ from $\hat{B}_{1min}$ to $\hat{B}_{1max}$, respectively.

In the second stage, the residual M_Z_/M_0_ following a train of hard pulses is approximated as a product of multiple cosines, which assumes on-resonance, perfect spoiling, and no T_1_ relaxation. The peak residual |M_Z_/M_0_| was minimized over a range of $\hat{B}_{1}$ values by varying an unordered set of flip angles using a Nelder-Mead numerical minimization subroutine [1] in MATLAB (R2013a; The MathWorks, Natick, USA).

In the third stage, off-resonance (B_0_) and T_1_ relaxation effects were considered by numerical solution of the Bloch equations using MATLAB code available online at [http://mrsrl.stanford.edu/~brian/blochsim](http://mrsrl.stanford.edu/~brian/blochsim/). RF pulse flip angles were altered by varying their duration, using a fixed B_1_ strength for all pulses in order to achieve the largest possible excitation bandwidth for each pulse. In the on-scanner pulse sequence implementation, the maximum achievable B_1_ field strength was calculated and used for every subject. For numerical optimization simulations, the typical maximum B_1_ strength measured for each field strength scanner was used. Perfect spoiling was assumed, and thus the spoiler durations before the first RF pulse and after the last RF pulse were omitted. T_1_ recovery was simulated during all other spoiler durations and RF pulses.

The peak residual |M_Z_/M_0_| was minimized over a range of B_0_, $\hat{B}_{1}$, and T_1_ values by applying Nelder-Mead numerical minimization in MATLAB to the unordered set of flip angles from the second stage. In each iteration, all possible permutations of the flip angle set were evaluated using the Bloch equation simulator over a range of B_0_, $\hat{B}_{1}$, and T_1_ values and the lowest mean residual |M_Z_/M_0_| from all permutations was returned. Overall computation time was approximately 6 minutes on a 3.6 GHz quad-core Intel Core i7 personal computer for a 5-pulse train. MATLAB source code used available online at <https://bitbucket.org/kelvinc/pulsetrainopt>.

**References:**

1. Nelder JA, Mead R: **A Simplex Method for Function Minimization**. *Comput J* 1965, **7**:308–313.
