# Supplementary figures and images for: Saturation pulse design for quantitative myocardial T1 mapping
Source: J Cardiovasc Magn Reson. 2015 Oct 1;17:84. doi: 10.1186/s12968-015-0187-0 (PMC4589956; doi:10.1186/s12968-015-0187-0)

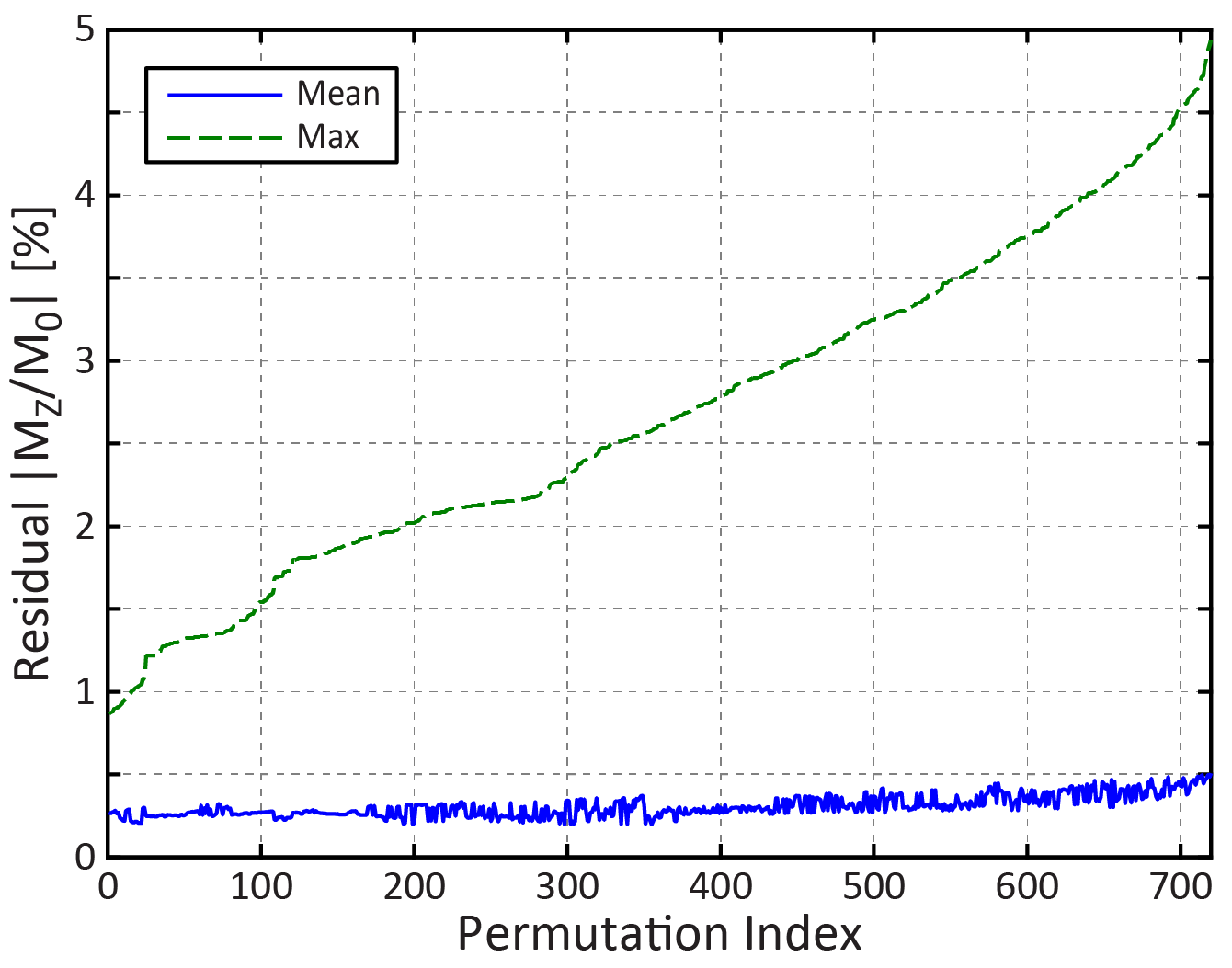

Supplement: Additional file 6: — Additional optimization code (MATLAB). (TIFF 4449 kb) [file 12968_2015_187_MOESM6_ESM.tif]
